# Identification and Characterization of Peanut Seed Coat Secondary Metabolites Inhibiting *Aspergillus flavus* Growth and Reducing Aflatoxin Contamination

**DOI:** 10.1021/acs.jafc.4c05517

**Published:** 2024-10-16

**Authors:** Leslie Commey, Yehia Mechref, Mark Burow, Venugopal Mendu

**Affiliations:** † Department of Plant and Soil Science, Texas Tech University, Lubbock, Texas 79409, United States; ‡ Department of Chemistry and Biochemistry, Texas Tech University, Lubbock, Texas 79409, United States; § Texas A&M AgriLife Research, Lubbock, Texas 79403, United States; ∥ Department of Agronomy, Agribusiness & Environmental Sciences, Texas A&M University, Kingsville, Texas 78363, United States

**Keywords:** aflatoxin, A. flavus, metabolomics, seed coat, liquid chromatography mass spectrometry (LC-MS), radial growth bioassay, secondary metabolites

## Abstract

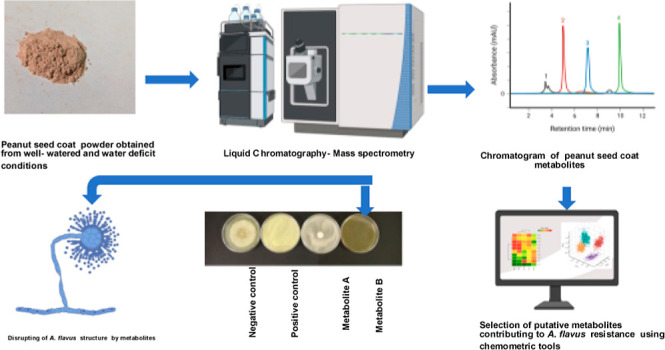

The peanut seed coat acts as a physical and biochemical barrier against *Aspergillus flavus* infection; however, the nature of the inhibitory chemicals in the peanut seed coat in general is not known. This study identified and characterized peanut seed coat metabolites that inhibit *A. flavus* growth and aflatoxin contamination. Selected peanut accessions grown under well-watered and water-deficit conditions were assayed for *A. flavus* resistance, and seed coats were metabolically profiled using liquid chromatography mass spectrometry. Kyoto Encyclopedia of Genes and Genome phenylpropanoid pathway reference analysis resulted in the identification of several seed coat metabolic compounds, and ten selected metabolites were tested for inhibition of *A. flavus* growth and aflatoxin contamination. Radial growth bioassay demonstrated that 2,5-dihydroxybenzaldehyde inhibited *A. flavus* growth (98.7%) and reduced the aflatoxin contamination estimate from 994 to 1 μg/kg. Scanning electron micrographs showed distorted hyphae and conidiophores in cultures of 2,5-dihydroxybenzaldehyde-treated *A. flavus*, indicating its potential use for field application as well as seed coat metabolic engineering.

## 1 Introduction

Peanut, also known as groundnut (*Arachis hypogea* L.), is an annual cash crop in the legume family grown mostly in arid and semiarid regions of the world for its high oil and protein content.^1^ As the majority of peanut acreage is grown without supplemental irrigation, peanuts are frequently exposed to unpredictable drought before harvest.^2^ Drought makes peanuts more susceptible to *Aspergillus* infection and aflatoxin contamination.^3^*Aspergillus flavus* and *Aspergillus parasiticus* are the main *Aspergillus* species that colonize peanuts and produce aflatoxin contamination either at the pre- or postharvest stage.^4^ Aflatoxin contamination in peanuts poses a significant food risk, causing adverse health effects such as abnormal function of the liver, kidney, and gastrointestinal tract.^5^ It is estimated that over 2 billion people in developing countries of sub-Saharan Africa and Asia are exposed to aflatoxin by unknowingly consuming aflatoxin-contaminated food that exceeds the European threshold of 4 μg/kg due to a lack of food safety inspection.^6^ In developed countries, despite the implementation of routine safety inspection, aflatoxin contamination remains a challenge; for instance, the US peanut industry loses peanuts worth up to $26 million annually due to aflatoxin contamination beyond the legal limit of 15 μg/kg.^7^ Thus far, physical, chemical, and biological strategies have been employed to address aflatoxin contamination in peanuts; however, these strategies have yielded limited outcomes.^8^ The use of resistant peanut varieties through breeding to control aflatoxin contamination seems to be the most effective approach; nevertheless, success has been slow due to the nonuniform distribution of aflatoxin contamination, variability of seeds to aflatoxin resistance, intricate genetics of aflatoxin resistance, and high environmental variability.^9^

After shelling, the peanut seed coat remains the only protecting layer against *A. flavus* colonizing the cotyledon. Research findings have demonstrated the influence of the physical and biochemical barrier provided by the peanut seed coat in inhibiting *A. flavus* infection.^10^ Nonetheless, how the seed coat can be deployed to manage aflatoxin contamination remains an important aspect that requires further investigation. The peanut seed coat serves as a reservoir for depositing secondary metabolites.^11^ These secondary metabolites are strategically deposited in plant tissues to defend against pathogens and abiotic stress.^12^ An array of secondary metabolites, including flavonoids, benzoic acid derivatives, hydroxycinnamic acid derivatives, coumarins, terpenoids, stilbenes, and phenolic compounds, has been identified as present in the peanut seed coat.^13^ A vast majority of these secondary metabolites are produced via the phenylpropanoid pathway.^14^

To identify the factors underlying resistance to *A. flavus* colonization within the peanut seed, genomic, transcriptomic, and proteomic approaches have been utilized to compare resistant and susceptible accessions. Genomic studies revealed a large number of differentially expressed genes (DEG) in the phenylpropanoid metabolic pathway of peanut seed when resistant lines were compared to susceptible lines upon infection with *A. flavus*.^15^ Transcriptomic and proteomic studies further attributed resistance to *A. flavus* infection to an increase in phenylalanine ammonia lyase, chitinase, and peroxidase transcripts,^16^ which leads to the activation of phenylpropanoid pathway metabolite-related proteins.^17^ Despite the significant progress, the correlation between *A. flavus* resistance in peanut seed and the identified transcripts and proteins remains unknown.

Metabolites are the final products that drive the expression of many genes and associated phenotypic differences.^18^ Metabolomic analysis has elucidated plant response to pathogens through plant–pathogen interactions.^19^ Metabolomics has led to the discovery of novel compounds associated with resistance to plant pathogens and human disease.^20^ Hence, the current study seeks to apply metabolomics to identify and characterize the peanut seed coat metabolites that inhibit *A. flavus* growth and aflatoxin contamination. In addition, this study investigated the metabolomic changes associated with drought, as they are related to *A. flavus* infection and aflatoxin accumulation. The outcome of this study provides a different approach to screen for *A. flavus*-resistant peanut varieties.

## 2 Materials and Methods

### 2.1 Chemical Reagents

3,5-Dihydroxybenzoic acid, 2,5-dihydroxybenzaldehyde, xanthohumol, naringenin, and daphnetin were purchased from AmBeed (Arlington Heights, IL, Illinois). Ferulic acid, *p*-coumaric acid, genistein, and nystatin were purchased from MP Biomedicals (Santa Ana, California). Potato dextrose agar (PDA), HPLC-grade acetonitrile, and formic acid were acquired from Fisher Scientific (Hampton, NH, USA). Eriodictyol was purchased from Thermo Scientific (Waltham, MA).

### 2.2 Fungal Inoculum Preparation

*A. flavus* toxigenic strains isolated and characterized using morphological, biochemical, and molecular methods^21^ were used for subsequent experiments. For inoculum preparation, an aliquot from the characterized *A. flavus* stock solution stored at −80 °C was added to a 100 mm Petri dish containing PDA media and was incubated in the dark at 28 °C for 8 days. 10 mL of distilled water containing 1% of Tween 80 (v/v) was added to the 8 day old culture to obtain a spore suspension for inoculum preparation. The spore suspension was filtered using sterilized cheesecloth, and the spore concentration was adjusted to 1 × 10^7^ cfu/mL with a hemocytometer (LW Scientific, Randall District, New York, US).

### 2.3 Plant Growth Conditions

Seeds from six peanut accessions BC_3_-60-02-03-02, BC_3_-43-09-03-02^22^ developed from a cross between Florunner^23^ and TxAG-6^24^ (a synthetic amphidiploid resulting from a three-way cross between the wild species *Arachis cardenasii*, *Arachis batizocoi*, and *Arachis diogoi*), PI 544346,^25^ 55-437,^26^ TMV-2,^27^ and Schubert^28^ were cultivated under both water-deficit (WD) and well-watered (WW) conditions. Experimental materials were planted in a randomized complete block design with five replications under WD conditions and three replications under WW conditions. More replications were used for WD to compensate for greater variability in yield compared to irrigated conditions. The WD experiment was conducted in a center-pivot irrigated field at the USDA-ARS Cropping System Research Laboratory in Lubbock, Texas. WD was initiated 52 days after planting (DAP) with a 25% evapotranspiration (ET) based on the West Texas Cotton model replacement maintained until harvest. The WW experiment was conducted in a flood-irrigated field at the Texas A&M AgriLife Research station in Lubbock, irrigated to 75% ET replacement. The seeds obtained from these two conditions were used for subsequent experiments.

### 2.4 Peanut Seed Grading

Pods obtained after harvest from WD and WW conditions were used for seed grading. For grading, 250 g of pods were randomly selected from three replicates of WW and WD conditions and passed through a riffler divider as described previously.^29^ Seeds were categorized into four seed sizes based on USDA official grading regulations for Spanish peanuts. The extra-large kernel (ELK) seed group was identified as seeds riding on a 25.4 mm × 8.5 mm (3/4 in. × 21/64″) screen. The medium seed category consisted of seeds that passed through the ELK screen but rode a 19.1 mm × 7.1 mm (3/4″ × 18/64″) screen. Number ones passed through larger screens but rode a 19.1 mm × 6.4 mm (3/4″ × 15/64″) screen. The other kernel (peewee) seed category was the intact seed that passed through all three screens.

### 2.5 In Vitro Seed Colonization Assay (IVSC) of Peanut Seed Grown under WD and WW Conditions

Graded seeds from the four categories of the six germplasm lines under WD and WW conditions were surface-sterilized using a 2% sodium hypochlorite solution and subsequently submerged three times in deionized distilled water to remove hypochlorite and any contaminating bacteria. Sterilized seeds were plated on a Petri dish with moist filter paper to provide favorable conditions for *A. flavus* growth. A 10 μL spore suspension (1 × 10^7^ cfu/mL) of *A. flavus* was inoculated onto individual seeds, following the procedure detailed previously.^21^ Inoculated seeds were incubated at 28 °C in the dark. Fungal growth and *A. flavus* colonization were observed after 8 days. The percentage *A. flavus* colonization was calculated using the formula^30^ Percentage colonization = (number of seeds colonized/total seeds) × 100.

### 2.6 Extraction and Preparation of Peanut Seed Coat Samples for Untargeted Metabolomic Profiling

Seed coats from peanut seeds acquired from all six accessions grown under WD and WW conditions were carefully removed by hand while wearing gloves. Peeled seed coats were ground into a fine powder, and soluble crude extractions were performed using the acetone, acetic acid, and water procedure.^21^ In summary, an SPEX SamplePrep Freeze Mill 6870 (Metuchen, New Jersey) was used to pulverize peeled seed coats. Triplicate samples of the fine powdered seed coats (1 g each) from all six peanut accessions under both conditions were placed in individual 15 mL centrifuge tubes. Subsequently, 10 mL of acetone/water/acetic acid (70:29.5:0.5 v/v/v) was used to extract the samples, with a second extraction performed using fresh solution. The mixtures were shaken for 3 h in an orbital shaker at 300 rpm, followed by an additional 12 h in darkness. After extraction, the soluble crude extract was centrifuged at 2000*g* for 10 min, after all three replicates, from the first and second extracts among the six samples, were combined. A Savant speed vacuum concentrator (Waltham, MA) was used to dry the soluble crude extracts from the triplicate samples. The dried samples were reconstituted in 1 mL of a 1:1 ratio of water/acetonitrile containing 0.1% formic acid.

### 2.7 Untargeted Metabolomic Profiling from Peanut Seed Coats Grown under WW and WD Conditions Using LC-MS

A Vanquish Ultra High-Performance Liquid Chromatography system from Thermo Scientific (San Jose, CA) was used for this study. Deionized water containing 0.1% formic acid was used as mobile phase A, and acetonitrile containing 0.1% formic acid was used as mobile phase B. An Acquity UPLC HSS T3 C-18 column (1.8 μm × 2.1 mm × 100 mm, 100 Å pore size) at a flow rate of 0.35 mL/min was employed for chromatographic separation. Initially, mobile phase B was used at 5% for 1 min and then increased to 15% over 5 min. Afterward, a gradient increasing to 30% of mobile phase B took place over 1.20 min, increased to 35% in a step gradient for 1.88 min, and then increased to 70% over 4.32 min. Lastly, the composition was returned to 5% of mobile phase B for 6 min. A Q Exactive HF-Orbitrap mass spectrometer (San Jose, CA) was used for untargeted metabolic profiling after chromatographic separation. This analysis utilized electrospray ionization in negative ion mode, with a spray voltage of −1.6 kV and the transfer tube set to 300 °C. Full mass spectra, ranging from 75 to 750 *m*/*z*, were acquired through the Orbitrap mass analyzer. The resolution was set at 120,000, with a 3 × 10^6^ AGC target, 200 ms injection time, and a mass accuracy of 5 ppm.

### 2.8 Data Processing

Compound Discoverer software version 3.1 (Thermo Scientific, San Jose, CA) was used to identify and quantify the metabolomics composition present in the samples. Freestyle version 1.4 (Thermo Scientific) and XCalibur 4.2 software (Thermo Scientific) were used at a mass tolerance of 5 ppm to confirm the retention time of the mass spectra. Additionally, a transition list was generated by using Microsoft Excel 365. The data from the Excel data sheet were used to compare metabolites between WD and WW conditions using the R statistical package TIDYR and DPLYR packages, version 4.2. Origin 2.0 (Origin Lab, Northampton) software was used for principal component analysis (PCA). Heat maps were generated using the R statistical packages GGPLOT2, READXL, and TIDYR version 4.2.

### 2.9 In Vitro Antifungal Activity of Peanut Seed Coat Metabolites against *A. flavus* Growth

Metabolites that showed a statistically significant difference in relative abundance and a fold change value greater than 1.5 when resistant peanut accessions were compared to susceptible peanut accessions between different irrigation conditions were selected. The selected metabolites were screened for their antifungal activity using a modified poison food technique.^31^ Selected metabolites were dissolved in 50% dimethyl sulfoxide (DMSO) in water solution to a final concentration of 10 mg/mL. Thereafter, 1 mL of each dissolved compound was added into individual 50 mL centrifuge tubes, with each concentration repeated in triplicate. Additionally, 15 mL of PDA medium were added to the centrifuge tubes, and the solution was carefully mixed, poured into separate Petri dishes, and allowed to solidify. A 10 μL spore suspension (1 × 10^7^ cfu/mL) of *A. flavus* was inoculated at the center of each Petri plate, followed by incubation at 28 °C. Nystatin (fungicide) served as the positive control and 50% DMSO in water was used as the negative control. After 8 days, when the negative control Petri plate was completely filled with mycelium, the diameter of the mycelial mat in each Petri plate was measured. The percentage of *A. flavus* inhibition was calculated using the formula described by Loizzo, Said, Tundis, Rashed, Statti, Hufner, and Menichini^32^
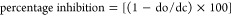
where do = diameter of the mycelium growth of the treated metabolites and dc = diameter of the mycelium growth of the negative control (50% DMSO in water). Minimum fungicidal concentration (MFC) is defined as the lowest concentration of an antimicrobial substance that results in the death of greater than 97% of fungal cells (Bhagwat & Datar, 2014). The MFC study used the metabolite that completely inhibited *A. flavus* colonization among the selected metabolites. Nine different concentrations (0.039, 0.078, 0.156, 0.313, 0.625, 1.25, 2.5, 5, and 10 mg/mL) were attained by weighing and dissolving the compounds in 50% DMSO. These concentrations were added into PDA media and inoculated with *A. flavus*, and fungal growth was observed after the radial growth bioassay. Nystatin was used at the same concentration as the positive control.

### 2.10 Estimation of Aflatoxin Contamination in Peanut Seed Coat Metabolites

For aflatoxin quantification, after the radial growth bioassay, PDA media containing metabolites and *A. flavus* growth were ground into a fine paste with a Kitchen Aid coffee grinder (Benton Harbor, MI). The resulting paste was dissolved in 100 mL of Vicam Aqua premix solution (Vicam Inc., Milford MA), centrifuged for 3 min to obtain a consistent mixture, and further filtered to produce a clear solution. To determine the aflatoxin concentration levels within the detection limit (2–100 μg/kg) of the Afla-V strips, we performed 10-fold serial dilutions based on the color of the solution. A reference color scale led to these dilutions: a tan solution indicated no dilution, while lighter to deeper shades of green suggested 1, 2, and higher dilutions, respectively. After dilution based on the solution color, 100 μL of each sample solution was pipetted onto Afla-V strips. The strips were incubated for 5 min and then analyzed using the Vicam Vertu reader to obtain readings for total aflatoxin (B_1_, B_2_, G_1_, and G_2_). PDA medium with no inoculation was used as a negative control check.

### 2.11 Effect of Peanut Seed Coat Metabolites on the *A. flavus* Fungal Hyphae Structure

The response of *A. flavus* morphology was observed for peanut seed coat metabolites used in the MFC study together with the negative control (PDA medium + 50% DMSO in water) after the radial growth bioassay using scanning electron microscopy (SEM). Mycelial blocks were precisely cut into 1 × 1 mm pieces from the edge of the culture media to prepare the samples. Samples were prepared using the procedure detailed previously.^33^ These steps involved chemically fixing the samples with a solution containing 2.5% glutaraldehyde in 0.05 M cacodylate buffer at 4 °C for 24 h. Subsequently, excess glutaraldehyde was removed from the samples by rinsing three times with a cacodylate buffer. After the initial fixation, samples were subjected to further fixation with 1% osmium-tetraoxide (OsO_4_) in 0.05 M cacodylate buffer for 1 h. Subsequently, samples were washed three times with 0.05 M cacodylate buffer to remove excess OsO_4_, with each wash done for 10 min for chemicals to soak and diffuse. The next step involved submerging the samples in water and rapidly freezing them in liquid nitrogen. After freeze-drying, samples were affixed onto aluminum sample mounts using double-sided carbon tape and then coated with a layer of iridium and carbon for SEM analysis. The SEM micrograph was observed using a Zeiss Crossbeam 540 (Dublin, CA).

### 2.12 Statistical Analysis

Statistical analysis was performed by PROC GLM using SYSTAT version 7.0 (SYSTAT Inc. Chicago, IL.) or SAS version 9.1 (SAS Institute, Cary NC). To study the influence of different peanut seed grades grown under different irrigation conditions on *A. flavus* colonization, seed size and irrigation conditions were treated as fixed effects, and replications were random effects. For the secondary metabolite experiment, the names of metabolites, irrigation, peanut accessions, and groups (cultivated resistant, wild species-derived resistant, and cultivated susceptible) were treated as fixed effects, and replication was a random effect. The expected mean squares and F test were calculated according to Schulz’s rules.^34^ Mean separation tests were performed using Fisher’s protected LSD test, with entries considered significant at *p* ≤ 0.05.

## 3 Results

### 3.1 Effects of Irrigation and Seed Grades of Peanut on *A. flavus* Colonization

**Figure 1:**
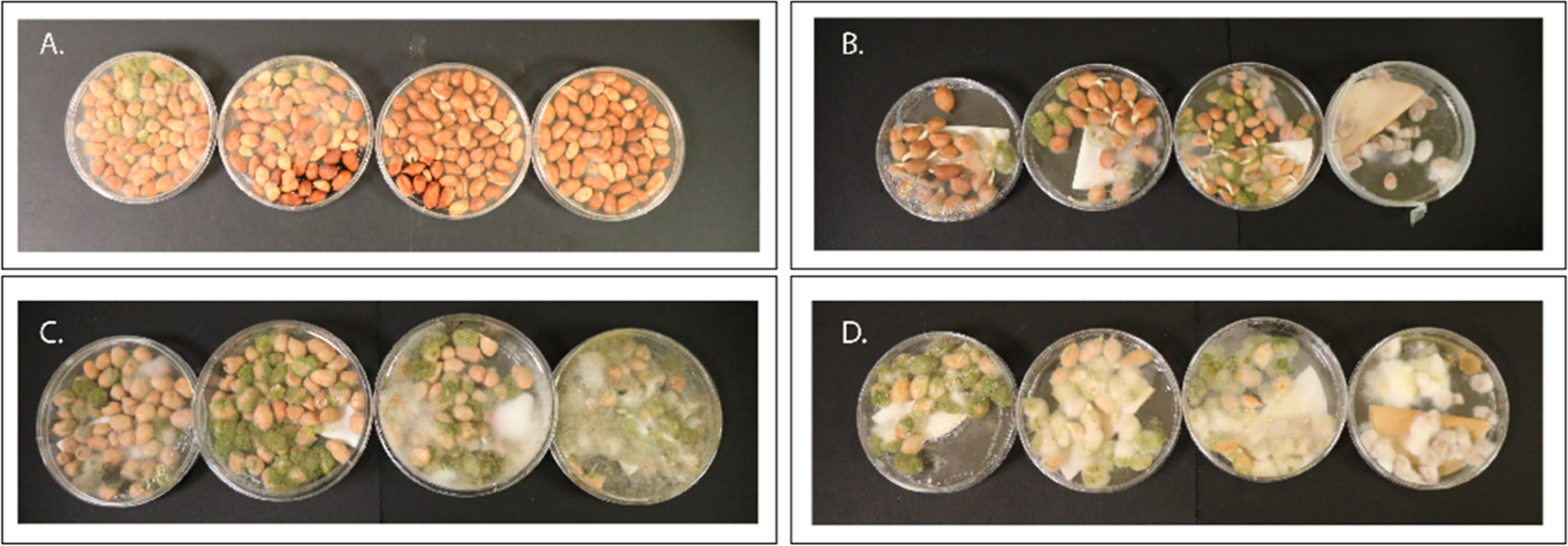
In vitro seed colonization (IVSC) assay of graded peanut seeds grown under WD and WW conditions. (A) *A. flavus* colonization level of PI544346 grown under WW conditions. (B) *A. flavus* colonization levels among different graded 55-437 peanuts grown under WW conditions. (C) Varying levels of *A. flavus* colonization among different graded PI544346 peanuts grown under WD conditions. (D) *A. flavus* colonization of graded Schubert peanut lines grown under WD conditions. For (A–D), the four plates are seeds of the four different seed sizes, from left to right: ELK, mediums, number ones, and peewees.

**Figure 2:**
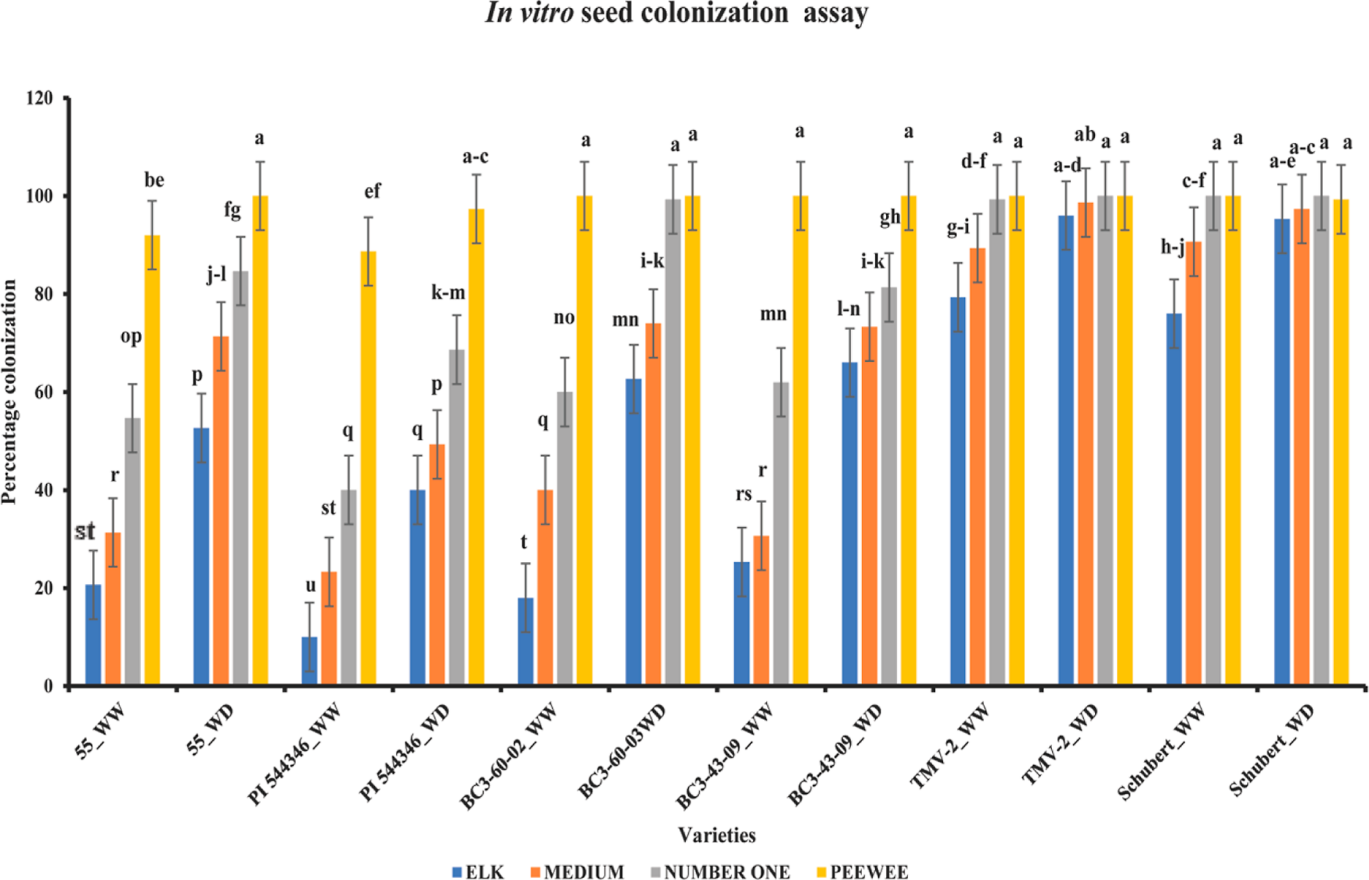
Percentage colonization of the six peanut accession seeds by *A. flavus* via the *IV*SC assay. The figure shows the percentage colonization of the different grades of peanut seeds grown under WW and WD conditions 8 days after the inoculation test. 55 is 55-437, BC_3_-60-02 is BC_3_-60-02-03-02, and BC_3_-43-09 is BC_3_ 43-09-03-02. Means not sharing the same letters are significantly different (*p* < 0.05) and the error bar represents the LSD value (*n* = 7.75).

*A. flavus* colonization rates were evaluated across different seed grades (sizes) of six peanut accessions grown under WW and WD conditions. The analysis of variance (ANOVA) results from the study revealed significant effects of irrigation, seed grade (size), and accessions on *A. flavus* colonization in peanuts (*p* < 0.0001) (Supporting Information for ANOVA, Table S1). Further analysis showed that the percentage colonization by *A. flavus* was higher for peanuts grown under WD conditions than under WW conditions (Figure 1). Among the four different seed grades, ELK had the lowest level of colonization by *A. flavus* under both irrigation conditions, while peewees showed the highest level of colonization (Figure 2). Among the six peanut accessions, based on the ELK seed grade and WW conditions, PI 544346 had the lowest percentage colonization (10%) followed by BC_3_-60-02-03-02 (18%), 55-437 (21%), and BC_3_-43-09-03-02 (25%) (Figure 2). Schubert and TMV-2 showed the highest rates of *A. flavus* colonization, 76% and 79%, respectively (Figure 2). Based on the data, PI 544346, 55-437, BC_3_43-09-03-02, and BC_3_-60-02-03-02 were identified as *A. flavus*-resistant, and Schubert and TMV-2 were identified as *A. flavus*-susceptible lines. The identified *A. flavus* resistant lines were further categorized as a cultivated resistant group (PI 544346 and 55-437) called group A and a wild species-derived resistant group (BC_3_-43-09-03-02 and BC_3_-60-02-03-02) called group B, based on their source. TMV-2 and Schubert were categorized as the cultivated susceptible group C. Overall, the data demonstrated that irrigation and grade of seeds influenced the susceptibility or resistance of peanuts to *A. flavus* colonization, with WD conditions and peewee seeds associated with high susceptibility to *A. flavus* colonization.

### 3.2 Identification of Metabolites Present in Peanut Seed Coat under WD and WW Conditions

**Figure 3:**
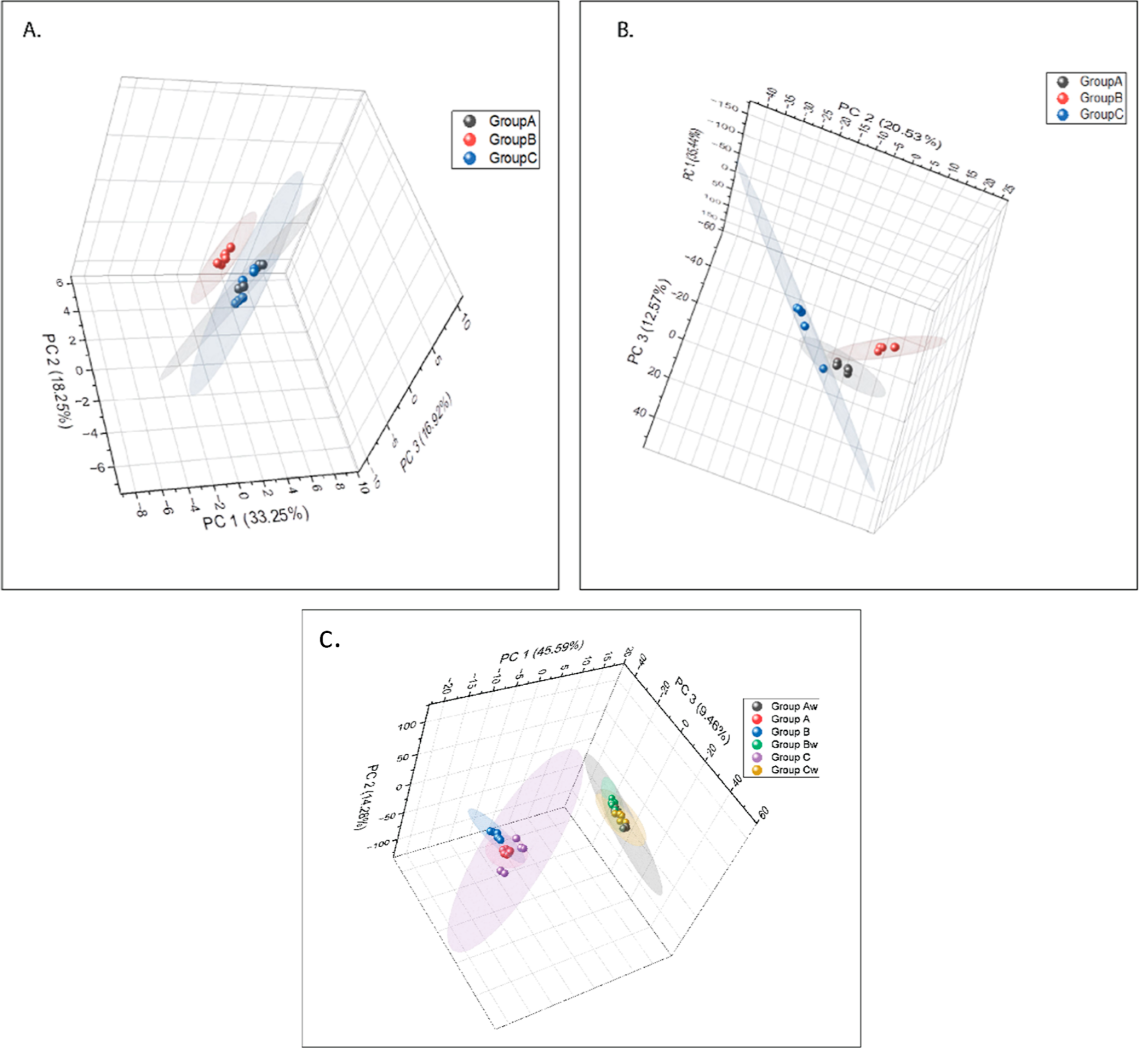
PCA of peanut seed coat metabolites specific to WD and WW conditions. Group A (black) consisted of cultivated *A. flavus*-resistant accessions (55-437 and PI 544346), group B (red) wild species introgressed *A. flavus*-resistant accessions (BC3-60-02-03 and BC3-43-09-02-03), and group C (blue) cultivated *A. flavus*-susceptible lines (TMV-2 and Schubert). Panel (A) represents the 172 peanut seed coat metabolites identified only from samples grown under WD conditions (R4 stage 57 DAP). Panel (B) represents the 517 peanut seed metabolites identified only from peanut samples grown under WW conditions. (C) PCA of peanut seed coat metabolites specifically common to WD and WW conditions. Group A (red) consisted of *A. flavus* cultivated resistant accessions (55-437 and PI 544346), group B (blue) wild species introgressed *A. flavus*-resistant accessions, and group C (violet) susceptible *A. flavus* accessions grown under WW conditions. Group Aw (black) consists of cultivated resistant *A. flavus* accessions (55-437 and PI 544346), group Bw (green) wild species introgressed resistant *A. flavus* accessions, and group Cw (yellow) susceptible *A. flavus* accessions, these three groups were grown under WD conditions.

The liquid chromatography mass spectrometry (LC-MS) study identified 914 metabolites under WW conditions; in contrast, 569 were identified under WD conditions (Tables S2 and S3). A comparative study of the metabolites identified under WW and WD revealed that 397 metabolites were common to both WW and WD conditions. Metabolites identified as unique to WW or WD conditions were 517 or 172, respectively (Tables S4 and S5). PCA was used to identify significant differences among the metabolites grouped into sets of common or unique to either WW or WD conditions among the three groups of peanut accessions [cultivated resistant (group A), wild species-derived resistant (group B), and cultivated susceptible (group C)] based on the *A. flavus* colonization. Significant differences were observed among the three groups of accessions based on the PCA, using the sets of 517 metabolites observed uniquely under WW conditions (Figure 3B).

By contrast, PCA of 172 metabolites unique to WD conditions did not show significant differences among all three groups; however, there were differences between group B and group A and also between group B and group C (Figure 3A). PCA using the set of 397 common metabolites showed significant differences between WW and WD conditions, but differences among all three groups of accessions were not as distinct (Figure 3C). To identify metabolites potentially contributing to *A. flavus* inhibition in peanut seed coats, the top 15 eigenvalues of principal component 1 from the PCA of both sets of common and unique WW conditions were selected (Table 1). Thirty different metabolites were identified as potentially explaining the phenotypic difference between resistant and susceptible peanut accessions. The 30 metabolites include sugars (3), carboxylic acids (3), imine (1), phenylpropanoids (6), fatty acids (5), benzoic acid derivatives (4), amino acid (1), terpenoid (1), glucoside (1), dipeptide (1), alkaloid (1), biphenyl (1), ester of coumarin (1), and glycolysis metabolites (1) (Table 1). The fatty acid metabolites include 9-hydroperoxyoctadeca-10,12-dienoic acid (9-HPODE), octanoyl glucuronide, 10-undecenoic acid, 1-palmitoylglycerol monoacyl glycerol (mag), and 9-hydroxy-10-undecenoic acid.

**Table 1:** Top Metabolites Identified Based on Eigenvectors from the PCA

	metabolites	formula	*m*/*z*	mass accuracy (ppm)	runtime	functional group
common	(*R*)-malate	C_4_H_6_O_5_	134.02149	–0.22	5.422	sugar
	2-hydroxy-2-butenedioic acid	C_4_H_4_O_5_	132.00589	0.15	4.239	carboxylic acid
	2,2-iminodipropanoate	C_6_H_11_NO_4_	161.06837	–1.241	0.935	imine compound
	2,3-dihydrogossypetin	C_15_H_12_O_8_	320.05233	–2.81	4.774	phenylpropanoid
	2,5-dihydroxybenzaldehyde	C_7_H_6_O_3_	138.03196	2.17	3.421	benzoic acid derivatives
	3-hydroxynonanoic acid	C_9_H_18_O_3_	174.12529	–1.72	9.477	carboxylic acid
	4-aminohippuric acid	C_9_H_10_N_2_O_3_	194.06873	–2.06	1.546	benzoic acid derivatives
	caffeic acid 3-glucoside	C_15_H_18_O_9_	342.09411	–2.63	4.277	phenylpropanoid
	chaparrin	C_20_H_28_O_7_	380.18254	–2.63	3.905	triterpenoid
	cinnamtannin A3	C_75_H_62_O_30_	1442.3272	–3.81	3.01	phenylpropanoid
	convicine	C_10_H_15_N_3_O_8_	305.08517	–2.62	2.275	glucoside
	d-serine	C_3_H_7_NO_3_	105.04278	1.9	0.875	amino acid
	glu-asp	C_9_H_14_N_2_O_7_	262.07921	–3.43	1.077	dipeptide
	octanoyl glucuronide	C_14_H_24_O_8_	320.14624	–2.81	6.295	fatty acid
	sebacic acid	C_10_H_18_O_4_	202.12013	–1.98	4.5	carboxylic acid
WW	(±)9-HpODE	C_18_H_32_O_4_	312.229	–3.2	14.443	fatty acid
	1,4-anhydro-6-*O*-dodecanoyl-2,3-bis-*O*-(2-hydroxyethyl)-d-glucitol	C_22_H_42_O_8_	434.28671	–2.76	11.756	sugar
	10-undecenoic acid	C_11_H_20_O_2_	184.14598	–2.17	10.021	fatty acid
	1-palmitoylglycerol; monoacyl glycerol (16:0)	C_19_H_38_O_4_	330.27635	–2.12	14.564	fatty acid
	2-succinylbenzoate	C_11_H_10_O_5_	222.05237	–2.25	3.922	benzoic acid derivatives
	3-(3,4-dihydroxyphenyl) pyruvate	C_9_H_8_O_5_	196.03678	–2.04	5.578	glycolysis
	3,5-dihydroxybenzoic acid	C_7_H_6_O_4_	154.02637	–1.95	2.867	benzoic acid derivatives
	3′,4′,5,6-tetrahydroxy-3,7-dimethoxyflavone	C_17_H_14_O_8_	346.06796	–2.02	4.106	phenylpropanoid
	4-methylumbelliferylacetate	C_12_H_10_O_4_	218.05737	–2.75	4.761	ester of coumarin
	5,5′-dehydrodivanillate	C_16_H_14_O_8_	334.06807	–2.39	2.899	biphenyl
	5,7-dihydroxy-2-(4-hydroxy-3-methoxyphenyl)-4-oxo-4*H*-chromen-3-yl 6-*O*-[(2E)-3-(4-hydroxyphenyl)-2-propenoyl]-beta-d-glucopyranoside	C_31_H_28_O_14_	624.1483	0.64	3.275	phenylpropanoid
	9-hydroxy-10-undecenoic acid	C_11_H_20_O_3_	200.14096	–1.5	9.057	fatty acid
	cassyfiline	C_19_H_19_NO_5_	341.1255	–2.34	9.349	alkaloid
	d-mannose 6-phosphate	C_6_H_13_O_9_P	260.03122	5.76	7.905	sugar
	ferulic acid	C_10_H_10_O_4_	194.05745	2.58	3.994	phenylpropanoid

### 3.3 Phenylpropanoid Pathway Metabolites Deposited in the Peanut Seed Coat

The eigenvalues in the PCA were generated based on the relative abundance of each metabolite in the entire set of metabolites. Due to their low relative abundance compared to other metabolites, only a few phenylpropanoid metabolites were identified among the 30 potential metabolites contributing to the inhibition of *A. flavus* growth on the peanut seed coat. To expand this number, we scanned the entire set of metabolites identified in the peanut seed coat. A total of 54 phenylpropanoid pathway metabolites were identified in the peanut seed coat under both irrigation conditions using the Kyoto Encyclopedia of Genes and Genome (KEGG) phenylpropanoid pathway as a reference. These metabolites belonged to anthocyanidin (2), flavonoid (10), flavanone (6), isoflavonoid (16), lignin (8), stilbene (4), lignan (3), and coumarin (5) biosynthesis pathways (Table 2). Flavanoids, flavanones, and isoflavonoids together form flavonoid metabolites.

**Table 2:** KEGG Reference Analysis of Phenylpropanoid Pathway Metabolites Present in the Peanut Seed Coat

metabolites	formula	*m*/*z*	mass accuracy (ppm)	runtime	phenylpropanoid pathway
cyanidin 3-*O*-(6-*O*-*p*-coumaroyl) glucoside	C_30_H_26_O_13_	594.13722	–0.17	2.944	anthocyanidin biosynthesis
cyanidin 3-*O*-sophoroside	C_27_H_30_O_16_	610.15428	1.31	5.226	anthocyanidin biosynthesis
catechin	C_15_H_14_O_6_	290.07815	–3.10	3.512	flavanoid biosynthesis
(+)-gallocatechin	C_15_H1_4_O_7_	306.073	–1.31	3.535	flavanoid biosynthesis
leucodelphinidin	C_15_H_14_O_8_	322.06794	–2.79	3.562	flavanoid biosynthesis
eriodictyol	C_15_H_12_O_6_	288.06278	–2.08	6.638	flavanoid biosynthesis
hesperetin 7-glucoside	C_22_H_24_O_11_	464.13082	–2.15	6.843	flavanoid biosynthesis
luteolin	C_15_H_10_O_6_	286.04686	–3.15	8.621	flavanoid biosynthesis
quercetin	C_15_H_10_O_7_	302.04166	–3.31	8.628	flavanoid biosynthesis
naringenin	C_15_H_12_O_5_	272.06764	–3.68	9.226	flavanoid biosynthesis
hesperetin	C_16_H_14_O_6_	302.07809	–0.33	9.646	flavanoid biosynthesis
xanthohumol	C_21_H_22_O_5_	354.14565	–3.11	13.605	flavanoid biosynthesis
(-)-epiafzelechin	C_15_H_14_O_5_	274.08419	0.26	4.835	flavanoid biosynthesis
rutin	C_27_H_30_O_16_	610.15278	–1.14	5.229	flavanone biosynthesis
quercetin-3β-d-glucoside	C_21_H_20_O_12_	464.09451	–0.15	5.588	flavanone biosynthesis
quercetin-3′-glucuronide	C_21_H_18_O_13_	478.07469	–0.21	5.663	flavanone biosynthesis
trifolin	C_21_H_20_O_11_	448.09955	–2.01	6.309	flavanone biosynthesis
luteolin 7-*O*-malonylglucoside	C_24_H_22_O_14_	534.10106	–0.19	6.907	flavanone biosynthesis
luteolin-6-C-glucoside	C_17_H_14_O_9_	362.06291	–2.21	3.459	flavanone biosynthesis
7-hydroxy-2′,4′,5′-trimethoxyisoflavone	C_18_H_16_O_6_	328.09382	–2.74	4.689	isoflavonoid biosynthesis
apigenin 7-(6″-malonylglucoside)	C_24_H_22_O_13_	518.10475	–2.32	7.11	isoflavonoid biosynthesis
(6a*S*,11a*S*)-3,6a,9-trihydroxypterocarpan	C_15_H_12_O_5_	272.06783	–2.21	9.113	isoflavonoid biosynthesis
2′-hydroxydaidzein	C_15_H_10_O_5_	270.05287	0.19	9.638	isoflavonoid biosynthesis
genistein	C_15_H_10_O_5_	270.052	–3.04	9.676	isoflavonoid biosynthesis
coumestrol	C_15_H_8_O_5_	268.03642	–2.98	9.786	isoflavonoid biosynthesis
2′-hydroxygenistein	C_15_H_10_O_6_	286.04793	0.70	9.852	isoflavonoid biosynthesis
(6a*R*,11a*R*)-3,9-dihydroxypterocarpan	C_15_H_12_O_4_	256.07304	–1.95	10.413	isoflavonoid biosynthesis
formononetin	C_16_H_12_O_4_	268.07289	–2.61	10.538	isoflavonoid biosynthesis
(-)-maackiain	C_16_H_12_O_5_	284.06854	0.11	11.587	isoflavonoid biosynthesis
glycitein	C_16_H_12_O_5_	284.06763	–3.06	11.607	isoflavonoid biosynthesis
2′-hydroxypseudobaptigenin	C_15_H_10_O_7_	302.04169	–3.64	6.953	isoflavonoid biosynthesis
(-)-glyceollin	C_20_H_18_O_5_	338.11556	0.59	10.315	isoflavonoid biosynthesis
(-)-vestitone	C_16_H_14_O_5_	286.08422	0.35	11.208	isoflavonoid biosynthesis
(-)-maackiain-3-*O*-glucosyl-6″-*O*-malonate	C_25_H_24_O_13_	532.12049	0.18	8.442	isoflavonoid biosynthesis
(-)-sophorol	C_16_H_12_O_6_	300.06257	–2.99	8.488	isoflavonoid biosynthesis
*p*-coumaric acid	C_9_H_8_O_3_	164.04714	1.22	3.683	lignin monomer biosynthesis
1-*O*-sinapoyl-beta-d-glucose	C_17_H_22_O_10_	386.12034	–2.6	3.833	lignin monomer biosynthesis
coniferin	C_16_H_22_O_8_	342.13051	–2.63	3.901	lignin monomer biosynthesis
caffeic acid	C_9_H_8_O_4_	180.04196	–2.22	3.991	lignin monomer biosynthesis
ferulic acid	C_10_H_10_O_4_	194.05745	–2.58	3.994	lignin monomer biosynthesis
sinapic acid	C_11_H_12_O_5_	224.06791	–2.23	4.048	lignin monomer biosynthesis
5-hydroxyconiferaldehyde	C_10_H_10_O_4_	194.05745	–2.58	4.32	lignin monomer biosynthesis
coniferylaldehyde	C_10_H_10_O_3_	178.0628	–1.12	5.253	lignin monomer biosynthesis
resveratrol	C_14_H_12_O_3_	228.07828	–1.75	8.687	stilbene biosynthesis
curcumin monoglucoside	C_27_H_30_O_11_	530.17786	–1.86	10.563	stilbene biosynthesis
curcumin	C_21_H_20_O_6_	368.12508	–1.36	12.031	stilbene biosynthesis
piceatannol	C_14_H_12_O_4_	244.07359	0.12	6.107	stilbene biosynthesis
(+)-pinoresinol	C_20_H_22_O_6_	358.14096	–1.67	11.265	lignan biosynthesis
(+)-syringaresinol	C_22_H_26_O_8_	418.16202	–1.91	9.851	lignan biosynthesis
lariciresinol	C_20_H_24_O_6_	360.15639	–2.50	7.979	lignan biosynthesis
5,6,7-trimethoxy-2*H*-chromen-2-one	C_12_H_12_O_5_	236.06795	–2.12	3.052	coumarin biosynthesis
daphnetin	C_9_H_6_O_4_	178.02632	–1.86	2.843	coumarin biosynthesis
fraxetin	C_10_H_8_O_5_	208.03671	–2.4	3.455	coumarin biosynthesis
pteryxin	C_21_H_22_O_7_	386.1357	–2.33	10.979	coumarin biosynthesis
scopolin	C_16_H_20_O_9_	356.10981	–2.53	6.896	coumarin biosynthesis

### 3.4 Identification of Putative Phenylpropanoid Pathway Metabolites Controlling *A. flavus* Resistance in the Peanut Seed Coat through Comparative Studies

**Figure 4:**
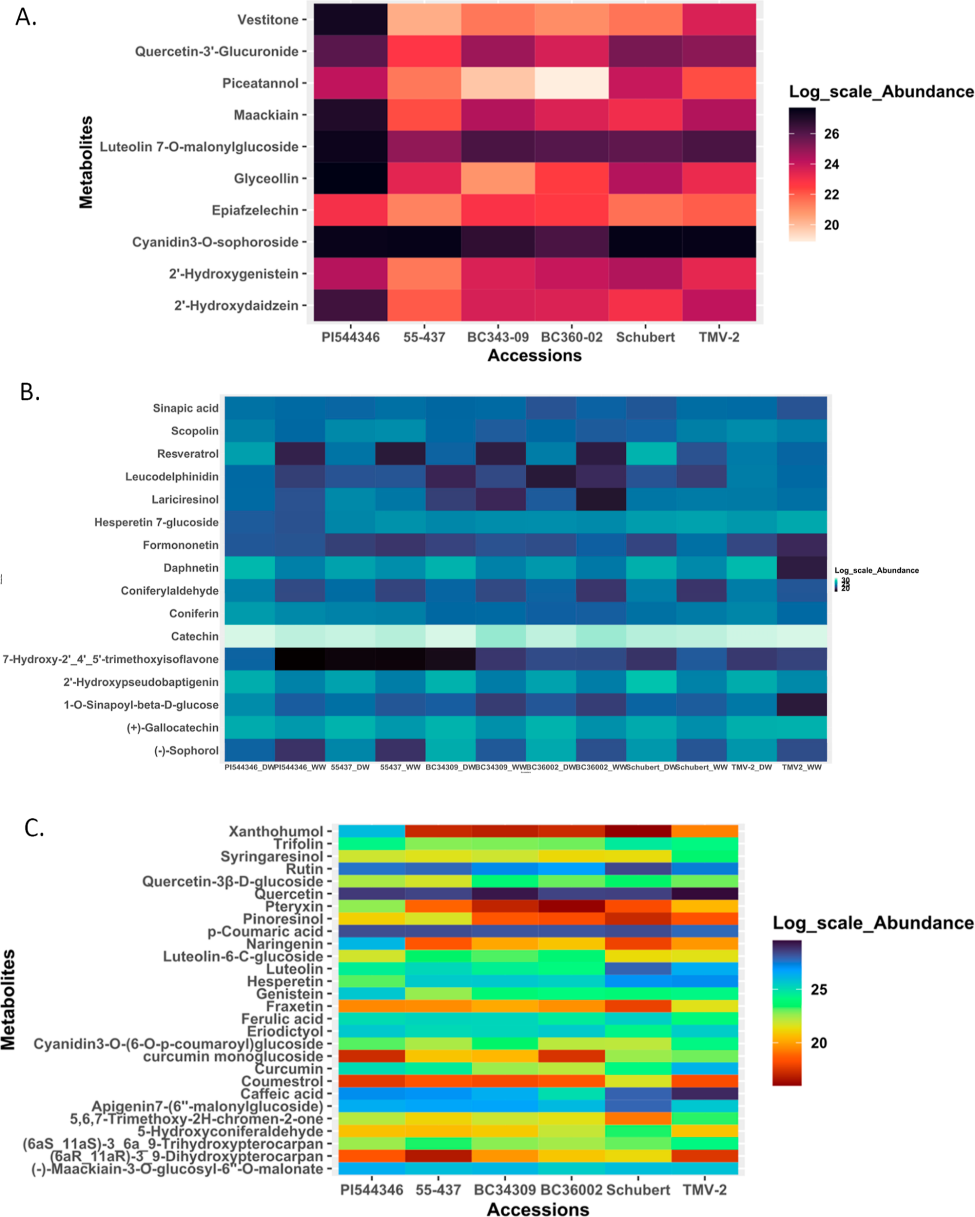
(A) Heat map of the relative abundance of phenylpropanoid pathway metabolites identified by KEGG analysis and present in the seed coat of peanut accession grown under WD conditions only. From the left is PI 544346 followed by 55-437 (group A), BC343-09 and BC360-02 are BC_3_-43-09-03-02 and BC_3_-60-02-03-02, respectively (group B), and Schubert and TMV-2 (group C). (B) Heat map of the relative abundance of the common phenylpropanoid metabolites present in peanut seed coat among peanut accessions grown under WD [DW] (57DAP) and WW conditions. From the left, PI 544346_DW, followed by PI 544346_WW, and the same pattern was done for the remaining 5 samples (55-437, BC_3_-43-09-03-02, BC_3_-60-02-03-02, Schubert, and TMV-2). (C) Heat map of the relative abundance of phenylpropanoid pathway metabolites present in the seed coat of peanut accession grown under WW conditions only. From the left is PI 544346 followed by 55-437 (group A), BC_3_-43-09-03-02 and BC_3_-60-02-03-02 (group B), and Schubert and TMV-2 (group C).

Among the 54 phenylpropanoid pathway metabolites identified in the peanut seed coat in the KEGG analysis, 28 were identified under WW conditions only (Figure 4C), with 10 being exclusive to WD (Figure 4A) and 16 present in both conditions (Figure 4B). The relative abundance of phenylpropanoid metabolites identified under three irrigation conditions (WW, WD, and both combined) showed a significant difference (*p* < 0.001) among the three groups of peanut accessions (cultivated resistant, wild species-derived resistant, and cultivated susceptible) (Table S6 ANOVA). The phenylpropanoid pathway metabolites identified under both conditions had a higher relative abundance under WD conditions than WW metabolites (Figure 4B).

By comparison of resistant and susceptible accessions, 17 out of the 54 phenylpropanoid pathway metabolites were selected as potential contributors to *A. flavus* resistance in the peanut seed coat. These include nine out of the 28 that were unique to WW conditions, four out of the ten that were exclusive to water-deficient conditions, and four out of the 16 that were common to both conditions. The selected metabolites exhibited a fold change greater than or equal to 1.5 and were statistically significant (Table 3). Within the selected phenylpropanoid pathway metabolites, naringenin and xanthohumol had the highest fold change difference in comparing the strongest *A. flavus*-resistant accession (PI 544346) to group C (TMV-2 and Schubert) accessions (Table 3).

**Table 3:** Fold Change of the 17 Putative Phenylpropanoid Pathway Metabolites Selected from the KEGG Pathway Analysis Contributing to *A. flavus* Resistance in Peanut Seed Coat^a^

conditions	metabolites	PI544346/Schubert	PI 544346/TMV-2	55-437/Schubert	55-437/TMV-2	BC34309/Schubert	BC34309/TMV-2	BC36002/Schubert	BC36002/TMV-2
WD only	epiafzelechin	2.6 ± 0.3*	2.0 ± 0.2*	0.8 ± 0.1	0.61 ± 0.2	2.4 ± 0.2*	1.9 ± 0.1*	2 ± 0.2*	1.6 ± 0.1*
	(-)-maackiain	15.3 ± 0.4*	6.2 ± 0.2*	0.6 ± 0.1	0.2 ± 0.1	2.5 ± 0.2*	1 ± 0.2	1.4 ± 0.1	0.6 ± 0.2
	2′-hydroxydaidzein	10.4 ± 0.3*	4.8 ± 0.2*	0.5 ± 0.2	0.2 ± 0.1	1.6 ± 0.8*	0.7 ± 0.1	1.5 ± 0.2*	0.7 ± 0.1
	luteolin 7-*O*-malonylglucoside	3.1 ± 0.3*	2.3 ± 0.4*	0.6 ± 0.2	0.4 ± 0.1	1.3 ± 0.3*	1 ± 0.2	1.1 ± 0.1	0.9 ± 0.1
WW only	luteolin-6-C-glucoside	1.4 ± 0.2	1.2 ± 0.1	4.3 ± 0.6*	3.7 ± 0.2*	3.7 ± 0.2*	3.2 ± 0.1*	5.4 ± 0.3*	4.7 ± 0.2*
	naringenin	298.9 ± 13.2*	80 ± 8.4*	1.5 ± 0.2*	0.4 ± 0.1	4.7 ± 0.4*	1.3 ± 0.2	7.2 ± 0.3*	2 ± 0.3*
	eriodictyol	2.5 ± 0.2*	1.1 ± 0.2	1.8 ± 0.3*	0.8 ± 0.2	1.9 ± 0.3*	0.8 ± 0.1	2.4 ± 0.4*	1.1 ± 0.2
	xanthohumol	1015.6 ± 17.8*	98.6 ± 12.3*	2.2 ± 0.2*	0.2 ± 0.1	1.86 ± 0.2*	0.18 ± 0.1	2.3 ± 0.1*	0.2 ± 0.1
	ferulic acid	0.8 ± 0.1	2.1 ± 0.3*	0.9 ± 0.2	2.4 ± 0.3*	0.7 ± 0.1	2.3 ± 0.1*	0.6 ± 0.1	1.5 ± 0.3*
	cyanidin 3-*O*-(6-*O*-*p*-coumaroyl) glucoside	2.1 ± 0.2*	0.5 ± 0.1	1.2 ± 0.1	0.3 ± 0.1	2.5 ± 0.1*	0.6 ± 0.2	1 ± 0.1	0.2 ± 0.1
	apigenin 7-(6″-malonylglucoside)	0.4 ± 0.1	1.7 ± 0.2*	0.4 ± 0.1	1.9 ± 0.2*	0.4 ± 0.1	2 ± 0.3*	0.3 ± 0.1	1.3 ± 0.5
	*p*-coumaric acid	1.0 ± 0.2	1.5 ± 0.1*	1 ± 0.3	1.5 ± 0.1*	0.9 ± 0.1	1.3 ± 0.2	0.9 ± 0.3	1.3 ± 0.1
	genistein	3.5 ± 0.3*	2.7 ± 0.2*	0.4 ± 0.1	0.3 ± 0.1	0.9 ± 0.1	0.7 ± 0.3	1.2 ± 0.1	0.9 ± 0.1
common	1-*O*-sinapoyl-beta-d-glucose_WD	5.2 ± 0.5*	2.3 ± 0.2*	1.6 ± 0.4*	0.7 ± 0.2	1.3 ± 0.1	0.3 ± 0.1	0.6 ± 0.2	0.3 ± 0.1
	1-*O*-sinapoyl-beta-d-glucose_WW	1 ± 0.2	22.3 ± 0.5*	0.8 ± 0.2	17.5 ± 0.2*	0.3 ± 0.1	5.4 ± 0.3*	0.3 ± 0.1	5.9 ± 0.6*
	coniferin_WD	5.8 ± 0.3*	2.5 ± 0.2*	2 ± 0.3*	0.9 ± 0.2	1.5 ± 0.2	0.3 ± 0.1	0.5 ± 0.2	0.2 ± 0.1
	coniferin_WW	1.8 ± 0.2*	3.6 ± 0.2*	1.2 ± 0.3	2.5 ± 0.1*	0.5 ± 0.1	1.1 ± 0.1	0.3 ± 0.1	0.6 ± 0.1
	formononetin_WD	7.8 ± 1.1*	0.2 ± 0.1	2.7 ± 0.8*	0.05 ± 0.01	6 ± 1.4*	0.06 ± 0.01	0.5 ± 0.1	0.02 ± 0.01
	formononetin_WW	0.4 ± 0.1	7.3 ± 0.1*	0.09 ± 0.001	1.6 ± 0.1*	0.3 ± 0.1	5.9 ± 0.2*	0.5 ± 0.1	10.4 ± 2.2*
	daphnetin_WD	0.9 ± 0.1	1.6 ± 0.2*	0.7 ± 0.1	1.2 ± 0.1	0.2 ± 0.1	0.4 ± 0.1	0.3 ± 0.1	0.6 ± 0.1
	daphnetin_WW	85.5 ± 0.9*	0.7 ± 0.2	90.1 ± 3.2*	0.7 ± 0.2	61.5 ± 2.1*	0.5 ± 0.1	153.4 ± 0.5*	1.2 ± 0.3

a Note: Data represent the fold change of the relative abundance compared between each other. WD = water deficit, WW = well-watered. * means significant difference.

### 3.5 Efficacy of Peanut Seed Coat Metabolites against *A. flavus* Growth and Aflatoxin Contamination

**Figure 5:**
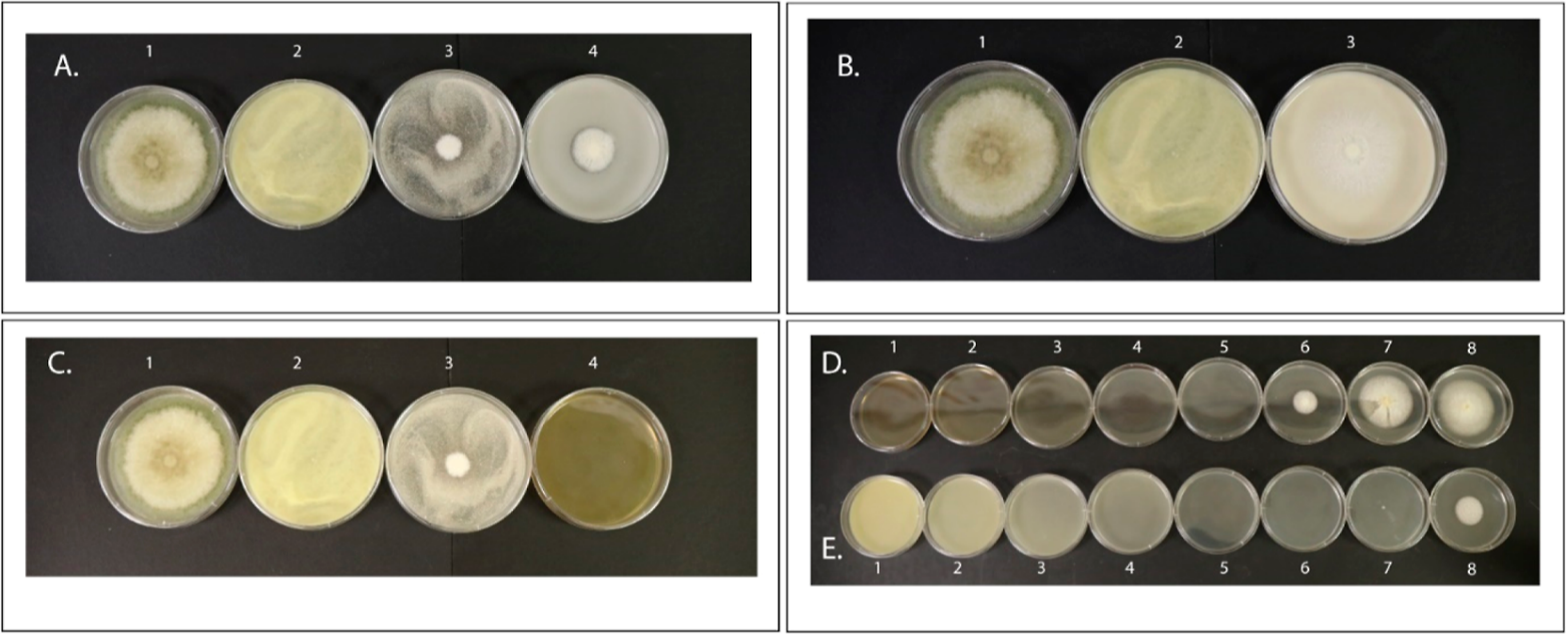
Antifungal activity of different peanut seed coat metabolites in response to *A. flavus* infection was assessed through the radial growth bioassay. (A1–C1) Radial growth of the negative control, consisting of 50% DMSO and water added to the PDA media. (A2–C2) Radial growth of *A. flavus* under positive control, a known fungicide (nystatin). Nystatin is typically yellow; the white appearance is due to its solubility in water and DMSO, with no *A. flavus* mycelium present. (A3,A4) Effect of ferulic acid and *p*-coumaric acid, respectively, on *A. flavus* growth. (B3) Radial growth of *A. flavus* when the media is amended with daphnetin. (C3,C4) *A. flavus* radial growth in the presence of ferulic acid and 2,5-dihydroxybenzaldehyde, respectively. (D,E) Identification of the MFC of metabolites present in peanut seed coat that inhibit *A. flavus* contamination, with (D1–D8) unveiling the response of 2,5-dihydroxybenzaldehyde and (E1–E8) showing the response of nystatin at eight different concentrations (10, 5, 2.5, 1.25, 0.625, 0.3125, 0.1563, and 0.0781 mg/mL) on *A. flavus* growth, respectively.

**Figure 6:**
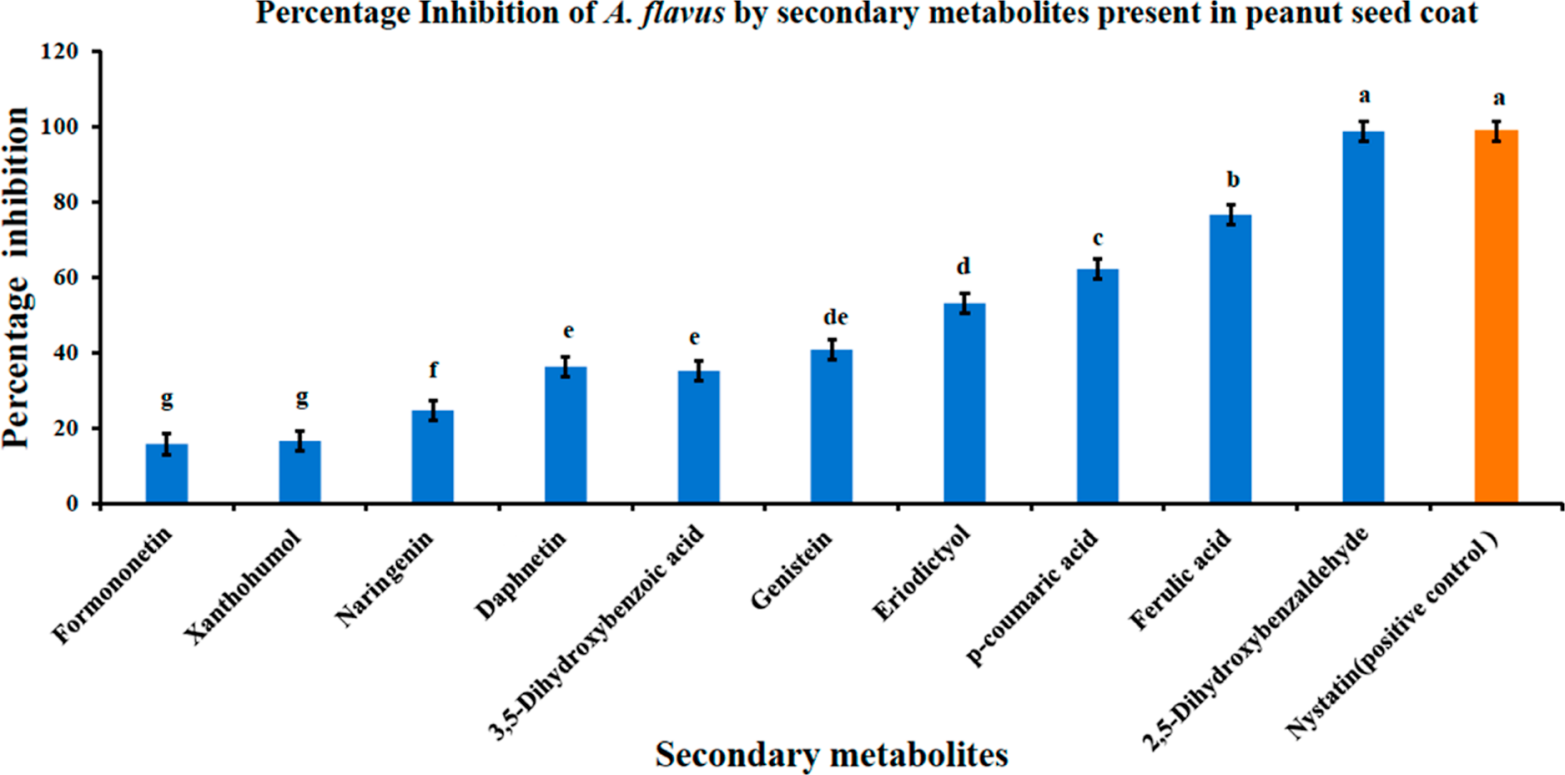
Inhibitory effect of secondary metabolites against *A. flavus* growth. The figure shows the percentage inhibition calculated after 8 days of the radial growth bioassay. ANOVA and LSD tests were performed to determine significant differences among the different metabolite inhibitions. Means not sharing the same letter are significantly different (*p* < 0.05) and the error bar represents the LSD value (*n* = 2.73) with three replicates each.

Seventeen phenylpropanoid pathway metabolites selected from the KEGG pathway analysis, together with 30 metabolites identified using PCA, yielded a total of 47 candidate metabolites potentially influencing *A. flavus* resistance in the peanut seed coat. Due to the constraints of availability and cost, we selected 10 out of the 47 metabolites for the radial growth bioassay (Table 4). Radial growth bioassays of these 10 selected compounds demonstrated that three metabolites reduced the mycelial growth of *A. flavus* by more than 50% at a concentration of 10 mg/mL (Figure 5). Nystatin and 2,5-dihydroxybenzaldehyde showed the highest *A. flavus* inhibition both reaching 98% (Figures 5 and 6). Ferulic acid and *p*-coumaric acid also significantly inhibited *A. flavus* growth with values of 76.5% and 62.1%, respectively (Figure 6). In contrast, formononetin (15.6%) showed the least inhibition together with the negative control, which had no inhibition of *A. flavus* growth. Xanthohumol and naringenin, which exhibited larger fold changes when *A. flavus*-resistant accession (PI 544346) was compared to susceptible accessions (TMV-2 and Schubert), showed lower levels of *A. flavus* inhibition with values of 16.5% and 24.7%, respectively. In terms of the selected metabolites’ ability to reduce aflatoxin contamination, ferulic acid- and *p*-coumaric acid-treated cultures had significantly lower aflatoxin levels (29.4 and 96.67 μg/kg, respectively) compared to the 994 μg/kg in the negative control. Nystatin and 2,5-dihydroxybenzaldehyde treatments resulted in 4 and 3 μg/kg aflatoxin, respectively (Table 5). The negative control check registered an aflatoxin contamination of 2 μg/kg (Table 5).

**Table 4:** Relative Abundance of the Ten Peanut Seed Coat Metabolites Selected for the Radial Growth Bioassay (×10^7^)^a^

metabolites	cultivated resistant		wild resistant		cultivated susceptible	
	PI 544346	55-437	BC36002	BC34309	Schubert	TMV-2
3,5-dihydroxybenzoic acid	113	160	130	219	49.7	443
ferulic acid	3.5	4.1	2.4	3.9	4.4	1.7
eriodictyol	5.2	4	4.8	3.9	2.1	4.5
*p*-coumaric acid	33	34	31	30.6	34.6	23.4
genistein	5.1	6.2	1.9	1.4	1.5	2
naringenin	74	0.3	0.2	0.1	0.02	0.08
xanthohumol	6.4	0.01	0.015	0.012	0.006	0.06
2,5-dihydroxybenzaldehyde_WW	55.4	59.9	42.4	71.8	8.4	34
2,5-dihydroxybenzaldehyde_WD	115	18.2	44.9	51.3	20.1	30.2
formononetin_WW	0.47	0.1	0.69	0.38	1.25	0.06
formononetin_WD	0.5	0.17	0.33	0.02	0.06	3.3
daphnetin_WW	24.9	4.5	1.8	2.7	3.8	0.003
daphnetin_WD	31.4	11	7.3	22	19	34

a Note WD = water-deficit, WW = well-watered.

**Table 5:** Estimation of Aflatoxin Contamination of the Ten Metabolites after Radial Growth Bioassay

metabolites	molecular formula	aflatoxin (μg/kg)
3,5-dihydroxybenzoic acid	C_7_H_6_O_4_	143 ± 7
ferulic acid	C_10_H_10_O_4_	30 ± 7
eriodictyol	C_15_H_12_O_6_	128 ± 10
formononetin	C_1_6H_12_O_4_	219 ± 8
*p*-coumaric acid	C_9_H_8_O_3_	97 ± 8
daphnetin	C_9_H_6_O_4_	302 ± 6
2,5-dihydroxybenzaldehyde	C_7_H_6_O_3_	3.1 ± 0.5
xanthohumol	C_21_H_22_O_5_	325.4 ± 6
genistein	C_15_H_10_O_5_	210 ± 5
naringenin	C_15_H_12_O_5_	218 ± 4
nystatin	C_47_H_75_NO_17_	4.7 ± 0.6
negative control		994 ± 3
control (PDA media no inoculation) check		2.3 ± 0.6

The radial growth bioassay examined eight different concentrations of 2,5-dihydroxybenzaldehyde in culture media and revealed that five concentrations of 2,5-dihydroxybenzaldehyde completely inhibited *A. flavus* growth, with the MFC observed at 0.63 mg/mL (Figure 5D). For nystatin, the MFC was observed at 0.31 mg/mL. The metabolite 2,5-dihydroxybenzaldehyde was identified under both WD and WW conditions, with a higher relative abundance in peanut accessions PI 544346 (115) and Schubert (20.1) under WD than under WW conditions [PI 544346 (55.4) and Schubert (8.4)] (Table 4). In contrast, the relative abundance of this metabolite decreased in the other four peanut accessions under WD compared to WW conditions. Among the six peanut accessions, the relative abundance of 2,5-dihydroxybenzaldehyde was higher in the cultivated resistant and wild species-derived resistant groups than in the cultivated susceptible group (Table 4).

To recap, several putative metabolites identified in peanut seed play a role in inhibiting *A. flavus* growth and contaminating it with aflatoxin. 2,5-Dihydroxybenzaldehyde emerges as a pivotal antifungal metabolite crucial in differentiating resistant from susceptible peanut varieties.

### 3.6 Effect of 2,5-Dihydroxybenzaldehyde on *A. flavus* Morphology and Structure

**Figure 7:**
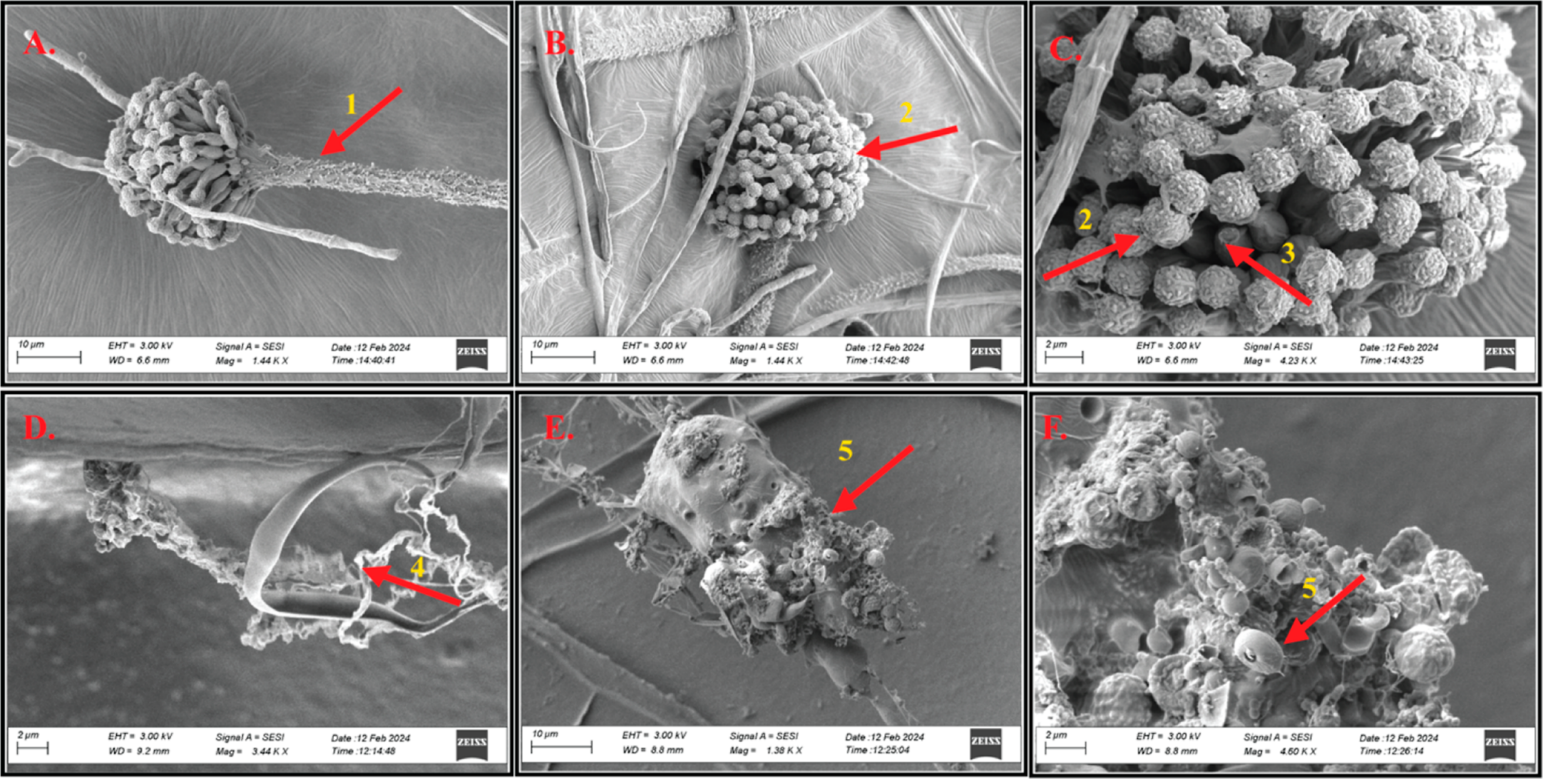
Scanning electron micrographs of the *A. flavus* structure of control and 2,5-dihydroxybenzaldehyde-treated plates after the radial growth bioassay. (A–C) *A. flavus* structure of the PDA medium treated with negative control (DMSO + water); (D–F) *A. flavus* structure of a PDA medium treated with MFC of 2,5-dihydroxybenzaldehyde after the radial growth bioassay. (A) Micrograph of an unchanged morphology of hyphae bearing a conidiophore; (1) detailed hyphae structure. (B) Multiple conidiophores bearing the conidia of *A. flavus* with no structural damage; (2) intact conidia structure. (C) Zoomed-in micrograph showing the phialides on the conidiophore; (3) intact phialide structure. (D) The micrograph shows disrupted, flat, and coiled hyphae; (4) hyphae structure. (E) The micrograph shows a distorted conidiophore with holes in them; (5) distorted conidiophore. (F) The micrograph shows distorted conidia with holes in them; (5) punched conidia.

We utilized SEM to visualize the structural changes induced by the 2,5-dihydroxybenzaldehyde treatment. We saw distinct changes in the fungal morphology between the untreated negative control (50% DMSO in water) and metabolite-treated cultures. In the untreated control, the distinct feature of the mycelium was a long, linear, tubular, and regularly spaced hyphal structure (Figure 7A), alongside intact conidiophores bearing well-conditioned conidia [Figure 7B (2)] and phialides [Figure 7C (3)]. The mature conidia were later dispersed, leading to the formation of new conidiophores. The fungal proliferation led to the expansion of the mycelial network, facilitating complete growth on the PDA medium (Figure 5A). Conversely, in mycelia treated with 2,5-dihydroxybenzaldehyde (0.63 mg/mL), significant morphological alterations were observed. Flat, coiled, and blistered hyphae were observed (Figure 7D) alongside disrupted conidiophores (Figure 7E) exhibiting holes for both conidiophores and conidia with deformed phialides that were not easily distinguished (Figure 7F). Overall, 2,5-dihydroxybenzaldehyde inhibited *A. flavus* colonization by altering the structures of hypha, conidiophore, and conidium, thereby reducing the ability of the fungus to proliferate and thrive.

## 4 Discussion

Secondary metabolites including low-molecular-weight molecules synthesized by the shikimate biochemical pathway and production of these compounds are influenced by the environmental interactions.^35^ Plants depend on certain secondary metabolites that are either constitutively present (phytoanticipins) or produced in response to a stress (phytoalexins) to withstand biotic or abiotic stresses.^36^ The peanut seed coat has been reported to serve as a reservoir for secondary metabolite deposition.^11^ We have recently reported the influence of peanut seed coat biochemicals in inhibiting *A. flavus* colonization.^21^ By using *IV*SC, previous studies^21,25,37^ and the current investigation confirm the resistance of PI 544346 and 55-437. Further, this study has established resistance of two BC_3_ interspecific introgressions lines BC_3_-60-02-03-02 and BC_3_-43-09-03-02 to *A. flavus* colonization with resistance derived from TxAG-6,^24^ one of the parents used in developing the BC_3_ lines. *A. cardenasii*, *A. batizocoi*, and *A. doigoi* (parents used in developing TxAG-6) were partially resistant to *A. flavus* colonization employing the *IV*SC method.^9^

This present study identified specific seed coat metabolites through LC-MS and a comparative examination of compounds in the phenylpropanoid pathway from *A. flavus*-susceptible and -resistant peanut accessions grown under WW and WD conditions. Ten of the putative metabolites revealed varying levels of inhibition to *A. flavus* growth in vitro, with 2,5-dihydroxybenzaldehyde being the most potent in inhibiting *A. flavus* by disrupting the reproductive and somatic structure of *A. flavus*. WD is an issue in peanut cultivated regions and a high percentage of *A. flavus* colonization observed on peanut accessions obtained from WD conditions suggests a potential role of water stress in *A. flavus* infection. This agrees with findings suggesting that WD is a predisposing factor for *A. flavus* colonization^37^ and is characterized by poor pod filling leading to a higher number of immature seeds with damaged pods, compromised seed coat, and breakdown of natural defense metabolites.^38^ Susceptibility of peanuts to *A. flavus* colonization increased as seed size decreased and agrees with several reports that demonstrated that *A. flavus* infection and aflatoxin contamination are consistently higher in smaller and immature kernels than in mature seeds.^39^ Sufficient irrigation during peanut production and elimination of small seeds and immature seeds from shelled peanuts could therefore play a role in managing *A. flavus* colonization and aflatoxin contamination. Overall, the current study has established different sources of *A. flavus*-resistant peanut accessions and the influence of seed size of shelled peanut and the role of WD on *A. flavus* colonization and aflatoxin contamination.

The low number of total and unique metabolites obtained in peanut accessions cultivated under WD conditions compared to WW conditions in this study may be attributed to the inability of metabolites to be mobilized and moved into the developing seed during the pod filling stage due to an insufficient amount of water.^40^ In correlation with our findings, a smaller number of peanut metabolites were identified in the drought-susceptible genotypes compared to the drought-tolerant genotypes in a metabolomic study.^41^ This implies that the presence or absence and relative abundance of metabolites between WW and WD irrigation conditions among the six peanut accessions could be used to select metabolites influencing *A. flavus* resistance in the peanut seed coat.

DEG, transcripts, and proteins linked with the phenylpropanoid pathway have been associated with resistance to *A. flavus* growth in peanut in genomic,^15^ transcriptomic,^16^ and proteomic studies.^17^ The presence of phenylpropanoid pathway metabolites has been established in the peanut seed coat via targeted metabolomics study with flavonoids emerging as the predominant metabolites.^13^ The current study identified similar phenylpropanoid metabolites in the peanut seed coat, and flavonoids were the most abundant phenylpropanoid metabolites. This validates the metabolites identified in this study to be truly present in the peanut seed coat. Additionally, PCA has been employed to identify potential metabolites contributing to disease resistance.^42^ Sugars, fatty acids, HPODE, and benzoic acid derivatives, together with phenylpropanoid pathway metabolites, were the different types of metabolites putatively attributed to *A. flavus* resistance in peanut seed coat in this work. The influence of these metabolites on the growth of *A. flavus* and aflatoxin contamination has been reported in previous studies. Effects include the maintenance of osmotic potential by sugars,^43^ reduction in fungal proliferation by coumarin,^44^ ferulic acid, a phenylpropanoid pathway metabolite,^21^ repression of aflatoxin production pathway genes facilitated by unsaturated fatty acid and, HPODE.^16^ Furthermore, benzoic acid derivatives have been reported to partially inhibit *A. flavus* resistance and aflatoxin contamination.^45^ Despite the putative influence of some metabolites on *A. flavus* growth, the metabolites that profoundly inhibit *A. flavus* remain mostly unknown.

The radial growth bioassay and the Vicam immunoassay revealed that the presence of 2,5-dihydroxybenzaldehyde, as an important metabolite in peanut seed coats, inhibits the growth of *A. flavus* and reduced aflatoxin accumulation when added to cultures. A previous study has demonstrated a similar observation of antifungal properties of 2,5-dihydroxybenzaldehyde against *A. flavus* strain NRRL 3357, *A. niger*, and *A. parasiticus* in a culture medium.^46^ The heightened antifungal efficacy of 2,5-dihydroxybenzaldehyde based on its chemistry has been postulated to stem from the specific arrangement of the aldehyde functional group in relation to the positioning of side groups on aromatic compounds.^47^ The observed SEM structural aberration in the somatic and reproductive structures of *A. flavus* is a result of the presence of 2,5 dihydroxybenzaldehyde and gives an indication of its biological effects on *A. flavus*. Similar structural aberrations have been reported on different fungi when treated with compounds identified to be antifungal and observed under the scanning electron microscope. This includes bioactive stilbenes on Botryosphaeriaceae^48^ and phenolic acids on *Fusarium oxysporum*.^49^ Interestingly, 2,5-dihydroxybenzaldehyde, also known as gentisaldehyde, is produced from the tyrosine metabolic pathway. This is the first metabolite identified in the tyrosine pathway to inhibit *A. flavus* growth and aflatoxin accumulation, in addition to the phenylalanine pathway which has been attributed to the growth inhibition of *A. flavus*.

To identify peanut seed coat metabolites that inhibit *A. flavus* and aflatoxin contamination, this study utilized *IV*SC to confirm the resistance of peanut accessions PI 544346 and BC_3_ lines (BC_3_-60-02-03-02 and BC_3_-43-09-03-02) to *A. flavus* colonization. Our non-targeted metabolomic analysis identified a novel peanut seed coat metabolite, 2,5-dihydroxybenzaldehyde, with potent antifungal activity against *A. flavus* growth and aflatoxin contamination (MFC of 0.63 mg/mL). SEM revealed that 2,5-dihydroxybenzaldehyde disrupts *A. flavus* reproduction and somatic structures, hindering its proliferation. Interestingly, the relative abundance of 2,5-dihydroxybenzaldehyde was higher in resistant peanut accessions under both WW and WD conditions compared to susceptible peanut accessions. These findings highlight 2,5-dihydroxybenzaldehyde as a promising antifungal agent and a potential biomarker for breeding resistant peanut varieties. Overall, this present study demonstrates the importance of exploring peanut seed coat metabolites as a strategy to manage *A. flavus* and aflatoxin contamination.

## Supporting Information Available

ANOVA of the percentage colonization of peanut seeds following the IVSC assay (Supporting Information Table 1); comprehensive list of WW conditions peanut seed coat metabolites identified under three distinct groups: group A (PI 544346 and 55-437), group B (BC3 60-02-03-02 and BC3 43-09-03-02), and group C (Schubert and TMV-2) (Supporting Information Table 2); total peanut seed coat metabolites identified under the same three groups (group A, group B, and group C) grown under WD conditions, with water stress imposed 52 DAP (Supporting Information Table 3); metabolites unique to WW conditions, determined by comparing metabolites identified under WW and WD conditions (Supporting Information Table 4); metabolites unique to WD conditions, determined by comparing metabolites identified under WW and WD conditions (Supporting Information Table 5); and ANOVA for phenylpropanoid metabolites identified using the KEGG phenylpropanoid pathway (Supporting Information Table 6). (XLSX)

## Abbreviations

LC-MS: Liquid Chromatography Mass Spectrometry
PCA: Principal Component Analysis
IVSC: *In**Vitro* Seed Colonization
KEGG: Kyoto Encyclopedia of Genes and Genome
CFU: Colony Forming Units
ET: Evapotranspiration Potential
DMSO: Dimethyl Sulfoxide
